# Massive Effect on LncRNAs in Human Monocytes During Fungal and Bacterial Infections and in Response to Vitamins A and D

**DOI:** 10.1038/srep40598

**Published:** 2017-01-17

**Authors:** Konstantin Riege, Martin Hölzer, Tilman E. Klassert, Emanuel Barth, Julia Bräuer, Maximilian Collatz, Franziska Hufsky, Nelly Mostajo, Magdalena Stock, Bertram Vogel, Hortense Slevogt, Manja Marz

**Affiliations:** 1 Friedrich Schiller University, Bioinformatics/High Throughput Analysis, Jena, 07743, Germany; 2 Jena University Hospital, Septomics Research Center, Jena, 07745, Germany; 3 FLI Leibniz Institute for Age Research, 07745 Jena, Germany; 4 Institute of Virology, Philipps-University Marburg, 35043 Marburg, Germany; 5 Chair of Bioinformatics, Friedrich-Schiller-University Jena, 07743 Jena, Germany

## Abstract

Mycoses induced by *C.albicans* or *A.fumigatus* can cause important host damage either by deficient or exaggerated immune response. Regulation of chemokine and cytokine signaling plays a crucial role for an adequate inflammation, which can be modulated by vitamins A and D. Non-coding RNAs (ncRNAs) as transcription factors or cis-acting antisense RNAs are known to be involved in gene regulation. However, the processes during fungal infections and treatment with vitamins in terms of therapeutic impact are unknown. We show that in monocytes both vitamins regulate ncRNAs involved in amino acid metabolism and immune system processes using comprehensive RNA-Seq analyses. Compared to protein-coding genes, fungi and bacteria induced an expression change in relatively few ncRNAs, but with massive fold changes of up to 4000. We defined the landscape of long-ncRNAs (lncRNAs) in response to pathogens and observed variation in the isoforms composition for several lncRNA following infection and vitamin treatment. Most of the involved antisense RNAs are regulated and positively correlated with their sense protein-coding genes. We investigated lncRNAs with stimulus specific immunomodulatory activity as potential marker genes: LINC00595, SBF2-AS1 (*A.fumigatus*) and RP11-588G21.2, RP11-394l13.1 (*C.albicans*) might be detectable in the early phase of infection and serve as therapeutic targets in the future.

Non-coding RNAs (ncRNAs) play essential roles in regulating protein-coding genes and gained an enormous medical relevance in the last decades. Although only less than 3% of the human genome codes for proteins^1^,^2^, it is believed that at least 80% of the human genome has a function^2^. Among these regions, which are mainly transcribed, the ncRNAs have been shown to regulate several basic cellular processes, such as transcription, processing, and translation. They are involved in chromosome structure, DNA replication, gene regulation, genome defense and protein transport. During the last decade, miRNAs received major attention as regulators of various cell processes. However, currently there are 2,468 ncRNA families described (Rfam v.12.0)^3^, of which 750 are known to exist in human, covering 1,853 genomic regions (http://rfam.sanger.ac.uk). Additionally, the number of long non-coding RNAs (lncRNAs, >200 nt, mostly with exon-intron structure) in humans is estimated to be 15,941^4^. They can be distinguished by their loci relative to protein annotations: intergenic, sense-intronic, sense-overlapping and antisense.

Vitamins A and D have been shown to play a regulatory role in immunity, prevent cancer development and can be used as therapeutical option in different malignancies^5^,^6^,^7^,^8^. However, the mechanisms have not been described so far. Although various publications report ncRNAs to be differentially expressed during infection diseases in human^9^,^10^,^11^, there is no knowledge about the role of ncRNAs during fungal infection and only a few studies have been reported about treatment with vitamins A and D. In the following, we summarize ncRNAs which have been brought in relation to vitamin supply or fungal infection.

## NcRNAs and atRA

All-trans-retinoic acid (atRA), the active metabolite of vitamin A, significantly regulates protein-coding genes and to an even higher fraction ncRNAs in human nCCIT cells^12^. In addition to micro RNAs (miRNAs) being regulated by atRA^13^,^14^, several lncRNAs have been reported to be influenced by atRA in neuronal and cancer cells. In rats, atRA is involved in hippocampal functions by up-regulating LINC_RBE^15^ and is able to restore NEAT1 expression in NB4 leukemia cell differentiation^16^. In the same cells and APL cell lines, a positive correlation of atRA and HOTAIRM1 has been shown. When HOTAIRM1 is regulated by atRA, less cell cycle genes are expressed, and genes involved in DNA replication as well as the expression of HOXD13 and HOXA11 are influenced^17^,^18^,^19^. Retinoic acid activates HOXD-AS1 (inducing the PI3K/Akt pathway) and the apoptosis repressor HOXA-AS2 in peripheral blood neutrophils^20^,^21^. Moreover, it has been shown that a reduced level of atRA leads to an induction of leukocyte activation, maturation and defense response^19^.

AtRA is also known to significantly down-regulate Malat1 expression during the differentiation of EML cells^22^. Furthermore atRA regulates LINC-RSAS in mammalian brain, which is associated with aging processes^23^ and lincRNA-EPS, which promotes terminal differentiation and inhibits apoptosis of mature erythrocytes^18^. In contrast to lncRNAs being regulated by various conditions, the retinoic acid signaling has been shown to be controlled by the lncRNAs LL18/NANCI and LL34, during endoderm development^24^.

## NcRNAs and vitamin D

Several lncRNAs have been reported to be affected by vitamin D in different cell lines. In mouse keratinocytes, a knockout of the the vitamin D receptor (VDR) leads to a down-regulation of tumor suppressor lncRNAs (Kcnq1ot1, lincRNA-p21, Foxn2-as, Gtl2-as, H10-as) and an increase in the expression of oncogenes (H19, HOTTIP, Nespas, mHOTAIR, Malat1 and interacting partner SRA)^25^,^26^.

VDR directly down-regulates miR-181a, co-regulates mir-106b, and finally leads to an up-regulation of p27. It targets mir-627 that in turn targets JMJD1A, miR-98 and let-7a-2. MiR-125b inhibits VDR expression and in turn the receptor down-regulates miR-125b^27^.

## NcRNAs during fungal infection

Although the knowledge regarding the regulatory impact of ncRNAs during fungal infection is limited, in *Arabidopsis thaliana* a few lncRNAs and lincRNAs have been shown to play an important role in antifungal immune response^28^. Also multiple miRNAs have been shown to be key elements in the respiratory epithelium during *Candida* infection^29^. The role of ncRNAs in humans during fungal infection and the influence of vitamins has not been described so far. On protein-coding level only few studies have been reported^30^,^31^.

As mentioned above, Vitamins A and D have been shown to play a regulatory role in immunity and preventing cancer where they have been used as therapeutic option^5^,^6^,^7^,^8^. However, the mechanisms have not been described so far. Here, we raise the hypothesis that ncRNAs might contribute to this effect.

In this paper, we find that vitamins A and D could directly regulate ncRNAs involved in immunity against fungal infections. We explore the effect of different pathogens on the expression of lncRNA in human monocytes. We describe specific effects during fungal infections when compared to bacterial infection with *E. coli*, which was used as outgroup in this study. Moreover, we explore the impact of atRA and vitamin D administration to obtain a systematic and comprehensive overview of vitamin regulated ncRNAs during infection. Many lncRNAs have been shown to be regulators of inflammatory chemokine and cytokine signaling. Using RNA-Seq we find both vitamins to regulate lncRNAs involved in amino acid metabolism and immune system processes, such as pro-inflammation and apoptosis. We present response specific and treatment specific results on different levels, for example single genes which are potential biomarkers and show structurally conserved regions and alternative splicing events.

## Materials and Methods

### Preparation of fungi and bacteria

Overnight cultures of *C. albicans* (SC5314) in YPD medium were washed three times with PBS and resuspended at 10^8^ yeasts/ml in RPMI 1640 GlutaMAX medium (Gibco, UK) supplemented with 10% fetal bovine serum (FBS; Biochrom, Germany).

*A. fumigatus* (AF293) was grown on AMM plates at 30 °C for 6 d. Conidiospores were harvested by rinsing the plates with water +0.05% Tween-20 (Sigma-Aldrich, Germany) and filtered through 70-*μ*m and 30-*μ*m pre-separation filters (Miltenyi Biotec, UK) to obtain a single-cell suspension. Conidia were then washed twice in PBS and resuspended at 10^7^ conidia/ml in RPMI 1640 GlutaMAX medium supplemented with 10% FBS (Biochrom, Germany). Germlings were obtained by incubation of conidia at 37 °C under continuous shaking for 6–8 h. They were then centrifuged and resuspended at 10^8^ cells/ml in fresh RPMI 1640 GlutaMAX medium supplemented with 10% FBS.

Overnight culture of *Escherichia coli* (isolate 018:K1:H7) in LB medium was washed three times in PBS and resuspended in RPMI 1640 GlutaMAX medium supplemented with 10% FBS. The concentration of bacteria was adjusted to 10^9^ cfu/ml. All pathogens were heat-killed by incubation at 65 °C for 30 min and immediately used for stimulation assays.

### Monocyte isolation

Human monocytes were isolated from 500 ml fresh whole blood (drawn within 1 h before use) of healthy male donors. Blood was layered onto an equal volume of 1-Step Polymorphs (Accurate Chemical & Scientific Corporation, USA) and centrifuged at 650 × *g* for 35 min. After centrifugation, the peripheral blood mononuclear cells (PBMCs) were collected, and normal osmolarity was restored by adding an equal volume of 0.45% cold NaCl. After erythrocyte lysis using a hypotonic buffer, cells were washed twice in cold PBS and counted using a Neubauer chamber. Cell viability of >95% was assessed by trypan blue staining. Monocytes were isolated from the PBMCs using the monocyte isolation kit II and quadro-MACS (Miltenyi Biotec, UK), following manufacturer’s instructions. Purity of the monocytes (>90%) was assessed as described in Klassert *et al*.^32^.

### Stimulation assays

Monocytes were resuspended at 5 × 10^6^ cells/ml in RPMI 1640 GlutaMAX medium (Gibco, UK) supplemented with 10% FBS (Biochrom, Germany) and 1% Penicillin/Streptomycin (Thermo Fisher Scientific, USA). They were seeded on 6-well plates (VWR International, Germany) and allowed to equilibrate at 37 °C and 5% CO_2_ for 2 h. Cells were then pre-incubated with 1 *μ*M atRA (Sigma-Aldrich (Germany) or 1*α*,25 (OH)2D3 (Sigma-Aldrich (Germany) for 30 min. Then, the heat-killed pathogens were added at a pathogen:host ratio of 1:1 for *C. albicans* yeast and *A. fumigatus* germ tubes, and 10:1 in case of *E. coli* stimulation. After 6 h of incubation at 37 °C and 5% CO_2_, cell viability >90% was assessed by trypan blue staining, and the monocytes were harvested for RNA isolation. The whole experimental workflow is depicted in Supplementary Fig. S1 by Klassert *et al*.^32^.

In total, we had four different immune-stimulatory settings (w/o infection, *A. fumigatus* infection, *C. albicans* infection and *E. coli* infection), in each of which we aimed to address the effect of vitamin A (atRA) or vitamin D supplementation.

### RNA sequencing

RNA was isolated from 5 × 106 monocytes using the RNeasy Mini Kit (Qiagen, Germany). An additional step was included to remove the residual genomic DNA using DNaseI (Qiagen, Germany). Quantity and quality of total RNA was assessed using a Nanodrop ND-1000 spectrophotometer (Thermo Fisher Scientific, USA) and a Tape Station 2200 (Agilent Technologies, USA), respectively. Strand-specific RNA libraries were constructed using the Dynabeads mRNA DIRECT Micro Purification Kit and the Ion Total RNA-Seq Kit v2.0 (Thermo Fisher Scientific, USA). Library templates were clonally amplified on Ion Sphere particles using the Ion PI Hi-Q Chef Kit and Ion Chef instrument (Thermo Fisher Scientific, USA), loaded onto Ion PI Chips and sequenced on an Ion Proton Sequencer (Thermo Fisher Scientific, USA). For sequencing, in total 48 samples were multiplexed on 12 chips. The raw sequence data in fastq format are stored in the Sequence Read Archive (SRA) at National Center for Biotechnology Information (NCBI) and can be accessed at NCBI homepage (accession number: SRP076532).

### Read preprocessing and rRNA filtering

Raw reads in fastq format were quality controlled with FastQC^33^ (v0.11.3) and trimmed with a window size of 10 using Prinseq^34^ (v0.20.3) (*Q* ≥ 20, *length* ≥ 20). The resulting reads were aligned to reference rRNA databases using SortMeRNA^35^ (v2.0). After quality control, up to 19 242 435 reads with a length of 20–373 bp and a GC content of ~50% were obtained for each sample (see Data availability section for external supplement S1).

### Mapping

The quality controlled and rRNA cleaned cleaned reads were aligned to reference genomes using Segemehl^36^ (v0.2.0). This mapping was performed against the human genome version GRCh38, downloaded from Ensembl (release 80). Indices were build together with the corresponding pathogen genomes, depending on the samples to be mapped. For *E. coli*, the complete genome of strain K-12 substr. MG1655 (NC_000913.3) was downloaded from NCBI. Genomes of *A. fumigatus* and *C. albicans* were obtained from aspergillusgenome.org (21.05.2015) and candidagenome.org (Ca21, 28.05.2015). All mappings were performed with default parameters and the –splits option of Segemehl to allow for multiple spliced read alignments.

### Differential gene expression

We used HTSeq-Count^37^ (v0.6.0) to quantify strand-specific and unique mapped reads on exon level. As reference, the full human Ensembl annotation (version GRCh38.80), including both protein- and non-coding genes, was used. Overall, 60 604 annotated features, comprising 19 825 protein-coding and 24 150 non-coding genes, were included in the annotation.

The raw read counts and rRNA cleaned read counts from each sample were normalized based on the library size and tested for significantly differentially expressed genes (adjusted p-value ≥ 0.05) using DESeq2^38^ (v1.10.1).

### Gene filtering

The DESeq2 results of different comparisons were additionally filtered by calculating TPM values for each gene in each sample as shown in ref. 39.


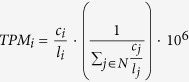


where *c*_*i*_ is the raw read count of gene *i, l*_*i*_ is the cumulative exon length of gene *i* and *N* is the number of all genes in the given annotation.

For each gene, we calculated four mean TPM values (*TPM*_*M*_), based on the 12 samples corresponding to control or one of the three different infection types. For protein-coding genes, we used a *TPM*_*M*_ > 5 to filter the final gene sets for further analyses. For ncRNAs, we used a minimum basemean read count of 10.

### Identification of different RNA isoforms

In order to identify different isoforms of antisense RNAs and lncRNAs, we investigated the expression patterns of the individual exons of these RNAs. Therefore, we adjusted our DESeq2 pipeline to calculate differentially expressed exons instead of whole genes Results were filtered by an adjusted p-value (≤0.05) and by basemean expression quantifier (≥40).

### Principal component analyses

Principal component analyses (PCA) were performed subsequent to the corresponding DESeq2 runs on selected gene subsets (e.g. only protein-coding genes or only lncRNAs) and with different variance cut-offs in R.

3-dimensional PCAs were plotted with the scatterplot3d package in R.

### Identification of co-regulated genes

To compare the effect of atRA and vitamin D during different infections, log2 fold changes (FC) as computed by DEseq2 were visualized using scatter plots in R.

The scatterplots were overlaid with contour plots for a two-dimensional kernel estimate (kde2d; MASS package) using the default parameters. Outliers were labeled with the respective gene names.

Box plots of certain gene expression patterns were visualized with the help of ref. 40.

### Heat maps

Gene-wise hierarchical clustering was performed on variance stabilized read counts to build heat maps of selected differentially expressed gene sets (adjusted p value ≤ 0.05). The input matrices for each heat map were scaled on rows to visualize changes in expression on gene level.

### Secondary structure prediction

The prediction of conserved secondary structures for potential marker genes is based on the multiz7way whole genome alignment, downloaded from UCSC (Octobre 2016) in multiple alignment format (MAF). Next to the human genome it contains the species *Pan troglodytes, Macaca mulatta, Canis lupus familiaris, Mus musculus, Rattus norvegicus* and *Monodelphis domestica*. According to the exons of human lncRNAs, alignments were sliced exon-wise and concatenated afterwards. To detect common secondary structures alignments were folded with RNAalifold (v2.1.8) from the Vienna RNA package^41^ following a window wise strategy (step width 50, window 300). Resulting structures were evaluated by hand.

### Ethics statement

The blood of healthy male donors was drawn after written informed consent. This is in accordance with the Declaration of Helsinki, all protocols were approved by the Ethics Committee of the University Hospital Jena (permit number: 3639-12/12).

### Data Availability

External supplement: http://www.rna.uni-jena.de/supplements/fungi_infection/.

## Results

The role of ncRNAs in humans during fungal infection and the influence of vitamins has not been described so far. Here, we investigate the transcriptional response of human monocytes after 6 h of infection with *A. fumigatus, C. albicans* and *E. coli* and, in addition, the influence of atRA and vitamin D during infection using RNA-Seq. We compared the three infections with control samples, as well as the three infections among each other. Furthermore, we determined the effect of vitamins in each type of infection. We particularly focused on antisense RNAs as potential cis-regulatory elements of related sense located protein coding genes and lincRNAs. Antisense RNAs and lincRNAs represent 92.2% of annotated lncRNAs and 56.2% of annotated ncRNAs in the human genome.

### Few highly expressed ncRNAs with strong effect in response to infection

**Figure 1:**
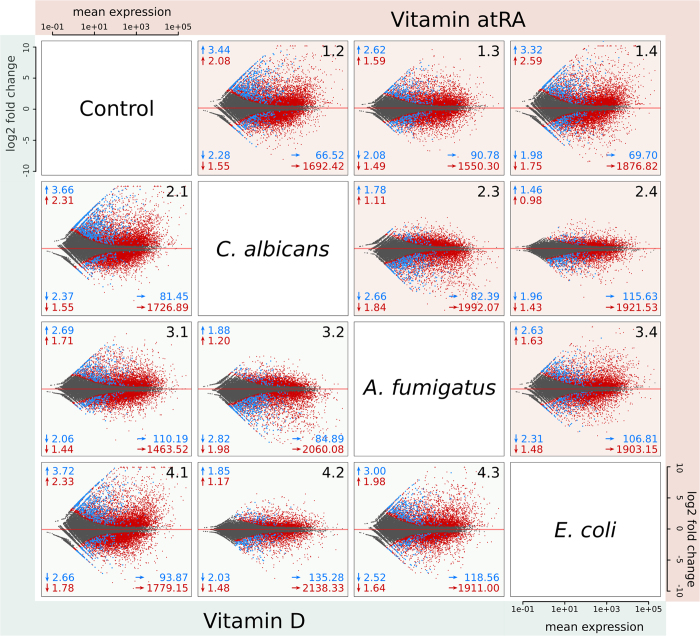
Low expressed ncRNAs with large fold changes. The overall effect and human gene signatures during pathogen infections upon atRA (upper right plots) and vitamin D (lower left plots) supply. Each subplot shows the mean expression of normalized read counts (x-axis, calculated by DESeq2) and log2 FC (y-axis) between two samples. Each dot corresponds to one gene. Colored dots show significant differentially expressed genes (red – protein-coding mRNAs, blue – ncRNAs) with an adjusted p-value ≤ 0.1. Mean positive and negative log2 FC are displayed in left corners, whereas the right bottom corners represent the mean normalized read counts. For example, the left scatter plot in first line shows more up- than down-regulated ncRNA genes during atRA-treated *C. albicans* infections as in the atRA-treated control sample. The second plot in the second line shows most genes being comparatively down-regulated in atRA-treated *A. fumigatus* infections compared to atRA-treated *C. albicans* infections, although *A. fumigatus* induces up-regulation of many genes in human monocytes (second plot in first line). Remarkably, ncRNAs are generally low expressed compared to proteins, but with large log2 fold changes.

We observed that about 5% of the lncRNAs and antisense RNAs were differentially expressed with high significance in at least one of the comparisons (see Table 1). In comparison to the 24% of highly significantly regulated protein-coding mRNAs^32^, the number of differentially expressed lncRNAs is low. However, these few ncRNAs are much stronger regulated than the protein-coding mRNAs (see Fig. 1; real fold changes up to 4000). Moreover, most of the strongly regulated ncRNAs are present in multiple related organisms with compensatory mutations to keep their potential secondary structures (see Data availability section for external supplement S10). This supports the hypothesis of a few strongly regulated ncRNAs to influence a large set of protein-coding genes.

**Figure 2:**
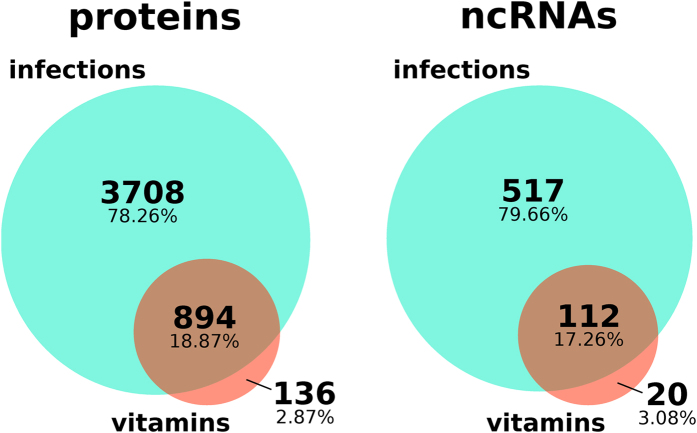
Less ncRNA genes affected by vitamins than pathogens. Number of significantly transcribed ncRNAs and protein-coding mRNAs during infection (no vitamin supply) and vitamin supply only. The ratio of vitamin- and pathogen-mediated regulation is similar between protein- and non-coding RNAs. Figure created using BioVenn^60^.

Interestingly, we see far more up-regulated than down-regulated non-coding RNAs during infection (see Fig. 1, plots in first column and first row). See also Klassert *et al*.^32^, where the number of protein-coding genes being up- and down-regulated are comparable. The expression pattern of ncRNAs in human monocytes during *C. albicans* and *E. coli* infection are similar (see Fig. 1, plot 2.4 and 4.2, with generally lower fold changes), whereas *A. fumigatus* infection shows a less intensive impact in human monocytes (see Fig. 1, plots 2.3, 3.4, 3.2 and 4.3). In general, the influence of vitamin supply on protein-coding mRNAs and ncRNAs is not as large as for the pathogens itself (see Fig. 2). At a first sight, the pathogen dependent transcription profiles upon vitamin A supply are comparable to those observed upon vitamin D supply. Nevertheless when we address the specific impact of both vitamins in each stimulatory setting, we could identify different patterns of vitamin dependent transcriptional regulation (see Data availability section for SFig. 4).

### Only few ncRNAs significantly expressed in response to atRA supply

**Figure 3:**
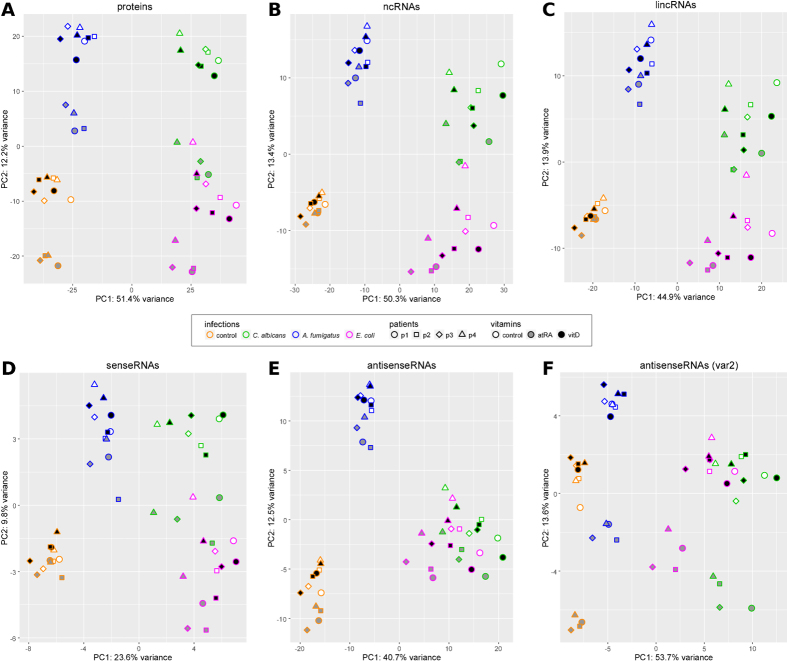
AtRA-treated samples show significant changes only among top antisense RNAs. Principal component analyses (PCA) based on top 500 variant genes for proteins (**A**), all ncRNAs (**B**), lncRNAs (**C**), sense RNAs (**D**), and antisense RNAs (**E**). (**F**) shows the 30 antisense RNA genes with a variance ≥2. Interestingly, atRA-treated samples show a significant change only among the top antisense RNAs. The top 30 regulated antisense RNAs and lncRNAs and their correlation are listed in heat maps as shown in the external supplemental material (see Data availability section). Different persons are labeled p1–p4.

Using principal component analysis (PCA) on the top variant 500 protein-coding and non-coding genes in the entire datasets, we were able to detect a strong impact of each pathogen. Nevertheless, the strong atRA specific effect observed in protein-coding mRNAs could not be observed for most of the ncRNA biotypes (see Fig. 3). This result is in line with Fig. 1, where we could observe only a modest regulation of ncRNAs when compared to protein-coding mRNAs during infection (see y-axis of Fig. 3A,B). For each subset of ncRNAs (lncRNAs Fig. 3C, sense RNAs Fig. 3D, and antisense RNAs Fig. 3E), we observe a similar pattern upon vitamin stimulation. We see only weak differences between control, vitamin D-treated and atRA-treated samples. We could only identify a slight atRA-dependent impact on antisense RNAs, which was consistent across all stimulatory settings (see Fig. 3E). This effect could be validated using a strict variance cutoff of ≥2 (see Fig. 3F), which reveals about 30 genes affected by atRA supply. About half of the 30 significantly expressed antisense ncRNAs are known to be related to immune response or infection and inflammation. Among them, we find AC073072.5 and RP11–536K7.5 to be antisense to the protein-based infection key player IL6 and receptor IL2RA, respectively^32^. Other antisense RNAs remain with unknown function.

With this analysis we are able to support the general assumption that a few regulatory ncRNAs are affected by treatment, but a tremendous number of protein-coding genes might be regulated by these few ncRNAs. One may also argue that only a few ncRNAs and an independent massive number of protein-coding genes are affected by atRA supply.

### Antisense RNAs involved in basic cell pathways

**Figure 4:**
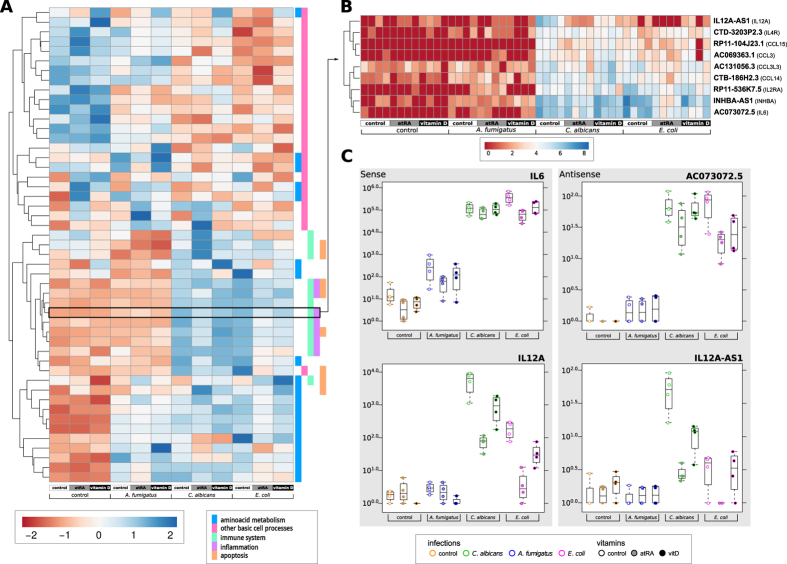
Expression analysis of antisense RNAs possibly involved in known pathways. (**A**) Pathway heat map of antisense read counts mapped onto the corresponding sense protein-coding genes as reported in KEGG with assigned ontology terms. (**B**) Heat map of antisense read counts mapped onto the corresponding sense protein-coding genes (written in parentheses) of the KEGG cytokine cytokine-receptor interaction pathway. Heat maps of all enriched pathways can be downloaded from the external supplement (see Data availability section for SFig. 7). (**C**) Box plots of the expression levels of *IL6* and *IL12A* as well as their antisense transcripts *AC073072.5* and *IL12A-AS1*, respectively.

The general function of antisense RNAs is unknown. However, they have been proposed to interact with the protein-coding mRNAs on the sensestrand^42^,^43^,^44^. Therefore, we used the potential sense mRNA-targets of all antisense RNAs, to explore their putative impact on known pathways. Expression data of antisense RNAs were artificially transferred to corresponding mRNAs. Pathway enrichment analysis of these protein-coding genes revealed several pathways which clustered together according to their ontology, thereby suggesting an impact of antisense RNAs on functions such as immune response, inflammation and apoptosis (see Fig. 4A). Interestingly, these biological functions were also highlighted by Klassert *et al*., when using the expression data of all protein-coding genes to perform gene ontology enrichment- and pathway analyses^32^.

**Figure 5:**
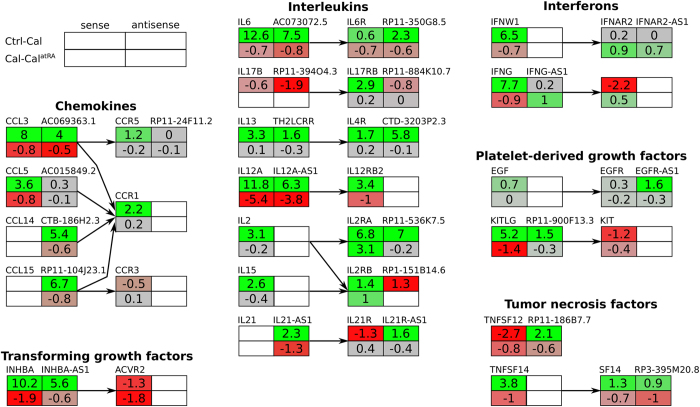
Cytokine antisense expression. Antisense expression of selected cytokines during *C. albicans* infection. Most antisense RNAs show differential expression patterns according to their sense protein coding genes. In particular, interleukins are strongly regulated.

We find antisense RNAs being generally down-regulated during infection with *C. albicans* and *E. coli*, but not for *A. fumigatus*. Protein-coding genes involved in amino acid metabolism mainly have antisense RNAs down-regulated in all tested infections, whereas antisense RNAs involved in other basic cellular processes seem generally up-regulated. For many antisense RNAs, we observe a surprising positive correlation to the expression levels of the sense mRNAs (see Fig. 5). Such a positive correlation of sense and antisense RNA transcription has been former reported^45^.

One of the significantly enriched regulated pathways from KEGG is the “cytokine-cytokine-receptor-interaction” pathway^46^,^47^. The most significantly involved antisense RNAs are displayed in Fig. 4B. Generally, we see only a marginal effect during *A. fumigatus* infection, in contrast to the dramatic up-regulation of cytokines and their receptors during *C. albicans* and *E. coli* infection. Also on single gene level we confirm a more similar reaction of monocytes to *C. albicans* and *E. coli* than between the two fungal pathogens. AC073072.5 (antisense to IL6) is more than 100 times up-regulated during *C. albicans* infection. Also, IL12A-AS1 is up-regulated during *C. albicans* infection. However, this effect is reduced by atRA supply.

In general, protein-coding genes for cytokines and their receptors, as described in ref. 32, are highly up-regulated during infection and down-regulated by atRA supply. Interestingly most of their antisense RNAs show a similarly strong behavior as described above for IL6 and AC073072.5 (see Fig. 5), which has never been reported in previous studies. However, a few antisense RNAs show an inverse transcription pattern as their corresponding cytokines, e.g. IL2RB–RP1-151814.6, IL21R–IL21R-AS1, and TNFSF12–RP11-186B7.7.

### Retinol metabolism modulated by atRA and vitamin D

**Figure 6:**
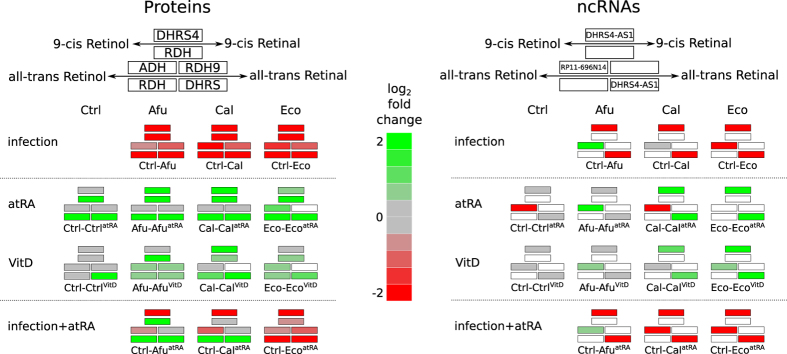
AtRA metabolism antisense genes influenced by pathogens. Differentially expressed protein coding genes (left) and their antisense ncRNA genes (right) involved in atRA metabolism. Human monocyte control samples (**Ctrl**) versus human monocytes infected with *A. fumigatus* (**Afu**), *C. albicans* (**Cal**) and *E. coli* (**Eco**) and/or treated with atRA (**atRA**) or vitamin D (**VitD**).

Since atRA has been shown to regulate immune response^32^, it is highly notorious that atRA-metabolism can be found among the top regulated pathways in our dataset (see Data availability section for SFig. 7). Therefore we investigated this pathway in more detail. We observed that the administration of atRA and vitamin D leads to an up-regulation of the enzymes SDR dehydrogenase/reductase member 4 and 9 (DHRS4,DHRS) as well as retinol dehydrogenase (RDH) and alcohol dehydrogenase (ADH) which are all responsible for the conversion of Retinol into Retinal as initial step for the generation of atRA. Interestingly, in absence of atRA supply, we can observe that pathogens inhibit the transcription of these Retinol metabolizing enzymes (see Fig. 6). Also the antisense RNA DHRS4-AS1 is down-regulated by pathogens, whereas upon vitamin supply a slight up-regulation can be observed. Thus, we observe a direct correlation between the antisense RNA and the DHRS gene family members. In contrast, the antisense RNA RP11-696N14 shows no consistent pattern suggesting an independent function. We conclude that atRA is able to regulate its own metabolism at transcriptional level, regulating not only protein-coding genes, but also antisense RNAs with putative functions in this pathway. The regulation of the retinol metabolism constitutes an additional example of counter-active effects between vitamin and pathogen stimulations.

### NcRNAs as potential marker genes

In our analysis, we find the ncRNAs known from the literature provided in the external supplement STable 3 (see Data availability section), to be involved in either fungal infections of plants or in human cancer development during atRA supply, to be only marginally affected. We find other previously annotated ncRNAs without an assigned function to be more relevant for fungal infection and upon vitamin supply. For a complete list of potential marker genes see Data availability section for external supplement STable 5). The function of novel ncRNAs is difficult to determine *in silico*. Secondary structures, with compensatory mutations and evolutionary conserved regions as potential protein interaction sites as provided in the external supplement S10 (see Data availability section). Genomically flanking (protein-coding) genes are described below to hint to a possible function.

**Figure 7:**
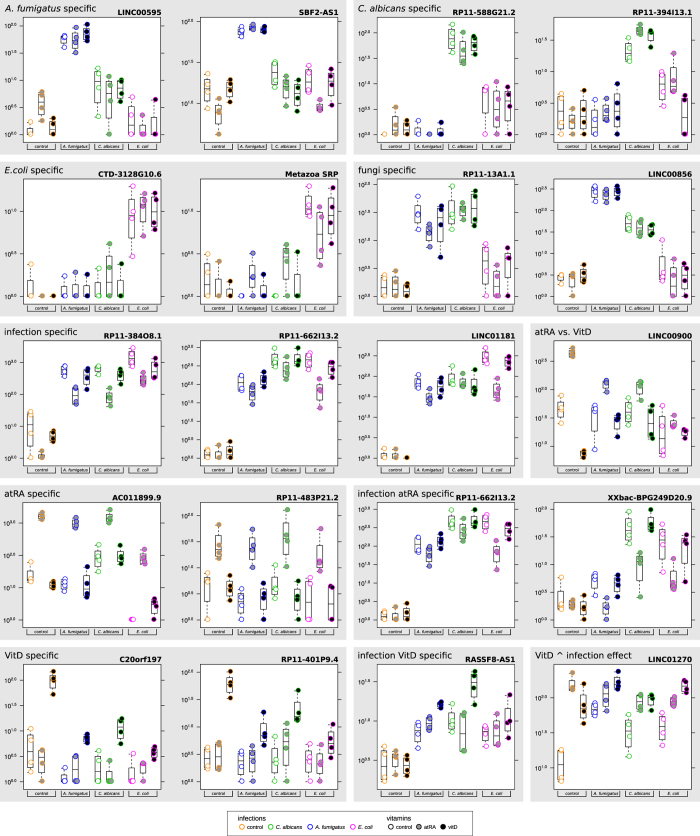
LncRNAs as potential marker genes. NcRNAs as potential marker genes for infections and vitamin supply. Lower right corner: vitamin D increases infection effect.

Figure 7 depicts ncRNAs as potential marker genes for various scenarios. The following functional assumptions are taken from GeneVisible, if not indicated differently. *A. fumigatus*-specific ncRNAs LINC00595 and SBF2-AS1 are described to be involved in inflammation and immune response. The first gene is located within a ribosomal protein cluster and near POLR3A, possibly associated with the translation machinery. The latter gene has been described just recently to promote proliferation in non-small cell lung cancer (NSCLC)^48^.

We identified RP11-588G21.2 and RP11-394I13.1 to be *C. albicans*-infection specific genes and CTD-3128G10.6 and SRP to be potential marker genes for *E. coli* infection. RP11-588G21.2 has been reported to possibly being involved in the maturation process of mature immune cells^49^.

SRP has been found in T-cell exosomes, suggesting that their amount might be increased during infection^50^.

Among the different infection types, we identified RP11-13A1.1 and LINC00856 to be specific for fungal challenge. For infections in general, we propose the following marker genes: RP11-384O8.1, RP11-662I13.2, and LINC01181. None of them has been reported previously to be of interest.

The ncRNAs AC011899.9 and RP11-483P21.2 appear to be specific for atRA supply (up-regulated by atRA in all stimulatory settings), whereas the infection-specific transcription of RP11-662I13.2 and XXbac-BPG249D20.9 are inhibited by atRA (without an effect in control samples). AC011899.9 and RP11-483P21.2 have previously only been reported to be a marker for mature beta-cells (like RP11-588G21.2)^49^. The concept of genes being up-regulated during infection and then inhibited by atRA supply has been also observed with protein-coding genes^32^ and these ncRNAs seem to follow this line.

The potential marker genes for higher vitamin D levels, C20orf197 and RP11-401P9.4, have been previously reported to be involved in tumorogenesis^51^. As one of only a few genes, we see LINC01270 being up-regulated during infection. Vitamin D supply increases this effect. LINC00900 is significantly up-regulated by atRA and down-regulated by vitamin D.

### Isoforms of antisense RNAs and lncRNAs during vitamin D supply

**Figure 8:**
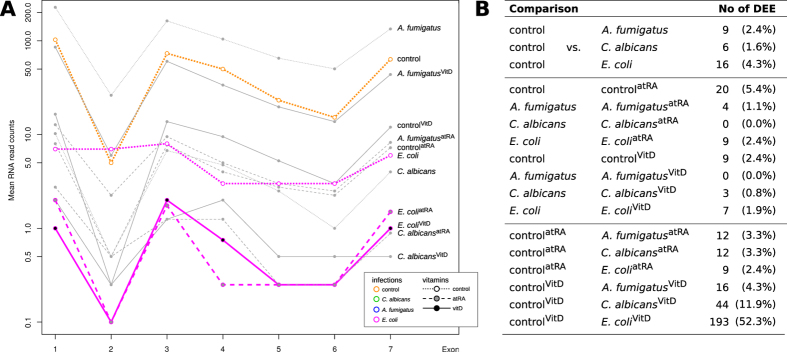
Differential expression of exons – isoforms of lncRNAs. (**A**) LncRNA AATBC. In most of the samples an isoform without exon 2 is generated (e.g. yellow control line). Upon *E. coli* infection most of the isoforms include exon 2, which can be reversed by application of atRA or vitamin D. (**B**) Overview of all differentially expressed exons from all lncRNAs. This analysis shows, that during infection and during atRA and vitamin D supply we see less differentially expressed exons compared to a combination of infection and vitamin supply.

Retinoic acid receptors (RAR) and retinoid X receptors (RXR) have three isotypes *α, β*, and *γ*, which are highly conserved between human and mice, suggesting specific functions for each receptor type^52^,^53^. LncRNAs can consist of several exons and undergo alternative splicing events leading to different isoforms^54^,^55^,^56^,^57^. These transcript variations may be expressed under various conditions, fulfilling a variety of different functions, similar to protein isotypes. We identified several alternative splicing events for antisense RNAs and lncRNAs within our samples (see STable 8). As one example, we found the lncRNA AATBC to show different isoforms during *E. coli* infection and treatment with vitamin D or atRA (see Fig. 8). AATBC has been recently described to be involved in suppression of cell proliferation, as well as apoptosis induction in bladder cancer^58^. Interestingly, many additional lncRNA isoforms were identified upon vitamin D treatment, especially during *E. coli* infection (see Fig. 8).

## Conclusion

Retinoids and vitamin D are known to play a regulatory role in development and physiology and are critical molecules in medical therapies. The mechanisms for this regulations are widely unknown. In this manuscript, we found lncRNAs to presumably contribute to their regulatory mechanisms. The number of differentially expressed lncRNAs is low compared to protein-coding mRNAs, but these lncRNAs are massively regulated (with fold changes higher than 4000). LncRNAs are regulated by atRA and vitamin D and are potentially involved in immunity, inflammation and apoptosis. The genes we have identified to be most important, and thus to be potential marker genes, have not been described previously and need further testing for validation of their putative function. We propose potential marker genes for infections in general and for specific fungal infections, which might be used as therapeutic targets in the future.

For the first time, the landscape of both the protein-coding mRNAs^32^ and non-coding RNAs was analyzed during fungal infection using high throughput sequencing of monocytes. Surprisingly, at ncRNA level, the immune responses during *C. albicans* and *E. coli* infection were more similar than during *A. fumigatus* infection. We revealed global systemic effects as well as detailed insights into the regulation of immune related proteins by lncRNAs, in particular antisense RNAs.

Although we did not find a single, but rather a set of key genes regulated by vitamins during infection, we could not identify common enrichments for response element motifs RARE (DR1, DR5) and VDRE in potential enhancer regions as reported before for proteins (see Data availability section for external supplement S10)^59^. However we demonstrate that vitamins counteract the transcriptional regulation of ncRNAs by *A. fumigatus, C. albicans* and the bacterium *E. coli*, thereby influencing the extent of the pro-inflammatory response. Cytokines, chemokines and apoptosis related antisense RNA genes are generally down-regulated by atRA. Retinoic acids are known to activate HOXD-AS1 (inducing the PI3K/Akt pathway) and the apoptosis repressor HOXA-AS2 in peripheral blood neutrophils^20^,^21^. We were able to verify these findings for human monocytes upon fungal infection shedding new light on lncRNAs. Furthermore, potential key marker genes in response to atRA include AC011899.9 and RP11-483P21.2 and RP11-588G21.2.

In conclusion, we have found several ncRNAs regulated by both vitamins during infection, which might play an important role regulating protein-coding genes. Moreover, we have identified potential marker genes for *A. fumigatus* (LINC00595, SBF2-AS1) and *C. albicans* (RP11-588G21.2, RP11-394l13.1) infections. The significance and fold change of expression of these genes is high enough, to assume their detectability in the early phase of fungal infection.

## Additional Information

**Accession codes:** The raw Ion Proton sequence data in fastq format are stored in the Sequence Read Archive (SRA) at National Center for Biotechnology Information (NCBI) and can be accessed at NCBI homepage (accession number: SRP076532).

**How to cite this article:** Riege, K. *et al*. Massive Effect on LncRNAs in Human Monocytes During Fungal and Bacterial Infections and in Response to Vitamins A and D. *Sci. Rep.* **7**, 40598; doi: 10.1038/srep40598 (2017).

**Publisher's note:** Springer Nature remains neutral with regard to jurisdictional claims in published maps and institutional affiliations.

## Figures and Tables

**Table 1:** Number of total and significantly differentially expressed lncRNAs, antisense RNAs and protein-coding mRNAs based on all pairwise sample comparisons.

	Total	A	B	C	C/Total
antisense	5561	1474	291	264	4.7%
lncRNA	7650	1457	394	321	4.2%
Protein coding genes	19825	14931	4038	4738	23.9%

Total – total number of human genes downloaded from ensembl.org v80; A – at least one read mapping to these genes; B – genes with variance >0.5; C – genes with an adjusted p-value < 0.05, absolute log2 FC > 2, and basemean read count >10. For an extended version of this table see Data availability section for external supplement (STable 9).
